# Therapeutic potential of CAR-T cell-derived exosomes: a cell-free modality for targeted cancer therapy

**DOI:** 10.18632/oncotarget.6175

**Published:** 2015-10-19

**Authors:** Xiang-Jun Tang, Xu-Yong Sun, Kuan-Ming Huang, Li Zhang, Zhuo-Shun Yang, Dan-Dan Zou, Bin Wang, Garth L. Warnock, Long-Jun Dai, Jie Luo

**Affiliations:** ^1^ Department of Neurosurgery, Taihe Hospital, Hubei University of Medicine, Shiyan, China; ^2^ Guangxi Key Laboratory for Transplantation Medicine, Institute of Transplant Medicine, 303 Hospital of People's Liberation Army, Nanning, China; ^3^ The Biomedical Research Center, University of British Columbia, Vancouver, Canada; ^4^ Department of Surgery, University of British Columbia, Vancouver, Canada

**Keywords:** immunotherapy, chimeric antigen receptor (CAR), exosomes, cancer therapy, extracellular vesicles

## Abstract

Chimeric antigen receptor (CAR)-based T-cell adoptive immunotherapy is a distinctively promising therapy for cancer. The engineering of CARs into T cells provides T cells with tumor-targeting capabilities and intensifies their cytotoxic activity through stimulated cell expansion and enhanced cytokine production. As a novel and potent therapeutic modality, there exists some uncontrollable processes which are the potential sources of adverse events. As an extension of this impactful modality, CAR-T cell-derived exosomes may substitute CAR-T cells to act as ultimate attackers, thereby overcoming some limitations. Exosomes retain most characteristics of parent cells and play an essential role in intercellular communications via transmitting their cargo to recipient cells. The application of CAR-T cell-derived exosomes will make this cell-based therapy more clinically controllable as it also provides a cell-free platform to diversify anticancer mediators, which responds effectively to the complexity and volatility of cancer. It is believed that the appropriate application of both cellular and exosomal platforms will make this effective treatment more practicable.

## INTRODUCTION

As a breakthrough in the year 2013, cancer immunotherapy marks a turning point in oncology treatment options [1]. In addition to drugs targeting immunologic checkpoint molecules such as CTLA-4, PD-1, and PD-L1, adoptive T-cell transfers as personalized targeted immunotherapy treatment option prevail in the war against cancer [2, 3]. Since the successful use of CAR-engineered T cells targeting CD19 molecules in patients with B-cell lymphoma was first reported in 2010 [4], immunotherapy has been moved from the sidelines of cancer treatment into the mainstream of modern oncology [5]. As a novel and potent therapeutic modality, there exists some uncontrollable processes which are the sources of adverse events, such as cytokine release syndrome (CRS), “on target, off tumor” response and life-threatening cytokine storms. Harnessing CAR-T cell-derived exosomes as ultimate attackers provides more control in key processes, thereby overcoming and stabilizing some of the therapeutic limitations in this model.

Exosomes are a sub-group of extracellular vesicles (EVs) which are secreted by most cells in the body. Based on their biogenesis, EVs are generally divided into three sub-groups including exosomes (30-150 nm in diameter), microvesicles (150-1000 nm) and apoptotic bodies (50-2000 nm). In recent years, cumulative evidence has emerged depicting the role of membrane vesicles as key mediators for intercellular communications. Of these vesicles, exosomes have received the most attention and have been well characterized. In the present review, we outline the general information of exosomes and CAR-based T-cell adoptive immunotherapy and focus on the therapeutic potential of CAR-T cell-derived exosomes in a cancer treatment model.

## OVERVIEW OF EXOSOMES

### Biogenesis of exosomes

Exosomes are nanoscale membrane vesicles (30-150 nm in diameter), first described by Rose Johnstone *et al* in the 1980s [6]. They originate from the endocytic compartment of the cells and are mainly composed of two parts, the round-shaped bilayer lipid membrane and the intravesicular content including membrane-anchored proteins [7]. The vesicular membrane is generated through two intervals of reverse invagination of the cellular plasma membrane. The first reverse budding takes place in the cellular plasma membrane, producing the early endosomes. The second reverse budding occurs on the limiting membrane of the late endosomes, which then develops multi-vesicular bodies (MVBs) while generating exosomal precursors known as intraluminal vesicles (ILVs) in the lumen of MVBs. The formation of ILVs is mediated by endosomal sorting complex for transport (ESCRT) machinery. Once ILVs are released into the extracellular space they are called exosomes. This process is achieved by fusion of the peripheral membrane of MVBs with the cellular plasma membrane. Apparently, outside-facing-out of the vesical membrane is ensured through the two intervals of reverse invagination of the plasma membrane. This is an essential prerequisite for exosomes to be applied for targeted cancer therapy because target orientation-related molecules from parent cells are also present in exosomes [8]. The intra-vesicular content is also closely related to the reverse invagination of the plasma membrane. At the MVB stage, the intraluminal content of nascent MVBs is equivalent to the extracellular milieu because the first reverse invagination takes place on cellular plasma membrane, whereas at the ILV stage, the intra-vesicular content is equivalent to the cytosol as the second invagination arises on the MVB membrane. Cytosolic components, such as microRNAs, mRNAs and proteins gain direct access to the interior of the forming vesicles during the generation of ILVs.

Exosomes are secreted through fusion of MVBs with the cellular plasma membrane. Many types of cells possess the capacity to release exosomes, including mesenchymal stem cells (MSCs)[9], dendritic cells (DCs) [10], B cells [11, 12], T cells [8, 13], NK cells [14] and tumor cells [15]. Exosomes are released from most donor cells constitutively, but their release is modulated by cell context. For example, human T cells secrete exosomes on the activation of T cell receptor (TCR) [8], DCs and B cells enhance exosome secretion following cognate T cell interactions [11, 16, 17].

### Composition of exosomes

The content of exosomes has been extensively analyzed through various techniques including PCR array, western blotting, fluorescence-activated cell sorting, mass spectrometry, antibody array and microarray. In addition to their spherical structure consisting of a lipid bilayer membrane, exosomes carry a complex cargo including nucleic acids, proteins and lipids. For example, using mass spectrometry, antibody array and microarray, Lai *et al.* have identified 857 unique gene products and > 150 microRNAs in MSC-derived exosomes [18, 19]. The exosomal proteins and microRNAs are implicated in various diverse biochemical and cellular processes. Exosomes have an evolutionary conserved set of proteins but they also have unique cell-specific proteins that vary depending on the cellular source and activation status [20]. Owing to their endosomal origin, exosomes typically do not contain mitochondria, endoplasmic reticulum or nuclear proteins. Nevertheless, exosomes contain a number of common protein components or house-keeping proteins that are necessary for the steady-state of the exosomal system and some of them can be used as common markers for exosomes [21]. According to their biological functions exosomal proteins are classified and summarized in Table 1.

**Table 1:** The functional classification of exosomal proteins

Biological function	Exosomal protein	Reference
Exosome biogenesis	ESCRT(I,II,III), Alix, TSG(10,101), GAG	[22-24]
Cytoskeleton organization	Actin, Adivillin, CAP1, Confilin, Moesin, Radixin, Talin, Tubulin	[25]
Tetraspanins (membrane protein organization)	CD9, CD37, CD53, CD63, CD81, CD82	[26, 27]
Transport and fusion	Annexins (I,II,IV,V,VI), RABGDI, RAP1B, RAB(5,7,11,27,35), Flotillin	[7, 21, 28-30]
Targeting/adhesion	Integrins(α_4_β_1_,αMβ_2_,αLβ_2_,β_2_), CD11_(a, b, c)_, MFG-E8/lactadherin, CD166,LFA-3/CD58, CD18, CD9, ALCAM, ICAM-1	[21, 28, 31]
Antigen presentation	MHC (I, II), CD86, HSC70, HSP84/90	[7, 32, 33]
Signal transduction	Gi2α/14-3-3, Gi3α, ERK2, FRL, Catenin, Fyn, Rho(A,C), GDI, Syntenin, G proteins	[25, 31]
Anti-apoptosis	Thioredoxine peroxidase, Alix, Galectin 3	[21]
Protection from lysis	CD55, CD59	[34]
Metabolic enzymes	ATP citrate lyase, ATPase, Thioredoxine peroxidase, Aldehyde reductase, Pyruvate kinase, GAPDH, AChE, Aspartate amino transferase, Fatty acid synthase, Glucose 6 phosphate isomerase	[7, 27]

### Transportation and biodistribution of exosomes

Exosomes are secreted from donor cells into the extracellular milieu through fusion of the peripheral membrane of MVBs with the plasma membrane. Once secreted, exosomes bind to neighboring cells or to the extracellular matrix, or passively transport through the bloodstream and other bodily fluids, such as lymph and cerebrospinal fluid. They are also present in many biological fluids including synovia fluid, breast milk, urine, saliva, amniotic fluid and malignant effusions of ascites. In blood serum, the density of total extracellular vesicles can be as high as 3,000,000 per microliter [35]. Exosomes have a very short half-life in the circulation *in vivo* and about 90% were cleared from the circulation within 5 min after injection [36]. The *in vivo* biodistribution of exosomes is determined by cell source, route of delivery and targeting condition [37]. In the recipient cells, intracellular uptake of exosomes takes place *via* membrane fusion, endocytosis, or receptor-mediated internalization [24]. As a result of their protein and microRNA composition which closely depends on the lineage and the state of activation, infection or transformation of the parent cells, exosomes can be applied for diagnosis, predicting prognosis and monitoring the response to treatment or progression of disease, simply through examining samples from blood or other bodily fluids [38-41].

### Biological functions of exosomes

Exosomes play an important role in intercellular communication [42]. Cell-cell communication can carry through receptor-mediated events, cell-cell synapses and direct cell-cell contact. Exosome-mediated communication between cells exhibits a new mechanism of cell-cell communication. Exosomes can act as shuttles for transferring microRNAs, mRNAs and proteins between cells [43]. This process may be occurring in the adjacent microenvironment, but could also take place at a distance by trafficking exosomes through the systemic circulation. Exosomes are even capable of passing through the blood-brain barrier [44-46]. Performing like a shuttle that carries cargo in its inner cabin, exosomes act as a one-way vehicle transporting various mediators through both intraluminal content and outer bilayer lipid membrane. In view of the fact that exosomes can deliver various signals simultaneously, an advantage of exosomes as vectors and mediators of intercellular communication is that the message can be targeted to multiple cells and multiple locations simultaneously. The general biological functions of exosomes were described in a number of reviews [21, 29], and some selected examples of cancer treatment-related functions are listed in Table 2.

**Table 2:** Cancer treatment-related functions of exosomes

Function	Molecule	Tissue or cell	Reference
Tumor antigen carrier	Tumor antigen	Cancer cells	[47, 48]
Antigen presentation	MHC/antigen	B cell	[49]
	MHC/antigen	DCs	[47, 50]
Directional cell motility	Syt7, R27a	Cancer cells	[51]
Immune suppression	IL-10, B7-H1	Cancer cells	[52, 53]
	miR-let-7b	T_reg_ cells	[13]
	IL-4	DCs	[54, 55]
	TGF-β	Cancer cells	[56]
Immune stimulation	MHC/antigen	DCs	[57, 58]
Immunotherapy	Tumor antigen	Cancer cells	[59, 60]
		DCs	[47, 50, 61]

## OVERVIEW OF CAR-T TECHNOLOGY

Adoptive transfer of chimeric antigen receptor-engineered T cells (CAR-T) suggests a promising new modality in the field of cancer immunotherapy. CARs are monoclonal antibody-based recombinant receptors that provide both antigen-binding and T-cell-activating functions. Once expressed in T cells, the CAR-T cells acquire potent antigen-targeted cytotoxic activity and act as “living drugs” applied as cancer therapy. Since the 1990's when CAR-expressing T cells were first evaluated as cancer therapy in preclinical experiments [62, 63], significant progress has been achieved. The results from early clinical trials have revealed a very encouraging therapeutic efficacy of CAR-mediated immunotherapy in a variety of cancers including acute and chronic lymphocytic leukemia [64, 65], lymphoma [66] and neuroblastoma [67]. CD19^+^ malignancies were the first cancers to be eliminated by CAR-engineered human T cells administered intravenously to tumor-bearing mice [68]. The first successful clinical use of CD19 CAR-T cells in a patient with B-cell lymphoma was reported in 2010 [4]. Additional reports confirmed the ability of these CD19 CAR-T cells to mediate the regression of B-cell lymphomas, and acute and chronic lymphoblastic leukemias [64, 69, 70]. CAR technology submissively imparts T cells supraphysiologic anticancer properties, which has been proved to be a pioneering breakthrough in the field of cancer therapy. This dramatic progress has repositioned and enhanced immunotherapy from the sidelines of cancer treatment into the mainstream of modern oncology [5]. However, as a new and developing therapeutic strategy, there exists few obstacles in the positive advancement of cancer immunotherapy. Currently, it is critical to properly handle CAR-T-induced adverse effects, such as “on-target, off-tumor” response and cytokine release syndrome (CRS) or cytokine storms, as some of these complications could threaten lives or lead to death. Another significant challenge is translating techniques so that this powerful therapeutic modality can be applied to treat solid tumors which account for over 90% of total cancers.

## THE ADVANTAGES OF USING CAR-T CELL-DERIVED EXOSOMES

### Exosomes as ultimate attackers making CAR-T treatment practically controllable

CAR-T cell-induced cytokine release syndrome (CRS) is one of the most adverse events that follows infusion of the CAR-expressing T cells [69, 71]. CRS is a disorder characterized by nausea, headache, tachycardia, hypotension, rash and shortness of breath caused by the release of cytokines from the immune cells. A cytokine storm is a severe form of CRS typically causing high fever and hypotension, potentially resulting in organ failure or even death [69, 71]. CRS occurs in nearly two thirds of CAR-T cell recipients and usually occurs within 10 days after cell infusion [71, 72]. Two deaths were reported during the early clinical trials with CAR-T cells [73, 74]. This type of life-threatening complication is mainly attributed to uncontrolled release of cytokines from CAR-T cells. First, the sudden outbreak of cytokines from a large number of infused antitumor lymphocytes (up to 10^11^) causes systemic inflammatory response similar to sepsis [3, 71]. A cytokine storm can be associated with arrhythmia, cardiac arrest, hepatic and renal failure thereby threatening the recipient's life. Second, CAR-T cells act as “living drugs” and can expand in an uncontrolled manner thereby inducing CRS. The influence of a costimulatory receptor such as CD28, 4-1BB, DPA10, OX40, or ICOS in the structure of second- and third-generation CARs not only elicits robust cytokine response but also enhances absolute T-cell expansion upon repeated exposure to antigens [75]. For example, the 4-1BB incorporated CD19 CAR-T cells can be expanded more than 1000-fold after administration in patients with chronic lymphocytic leukemia [64]. CAR-T cell expansion *in vivo* appears to be crucial and correlates with both response and toxicity. A number of medications have been used to effectively manage CRS-induced complications, including steroids, vasopressors, IL-6 blockade, and/or supportive therapy delivered in the intensive care unit [69, 70]. Unlike conventional drug-induced side effects, CAR-T cell-induced toxicity cannot be controlled by simply reducing drug dosage. Investigators even tried to include suicide genes in CARs as a safety switch to eliminate CAR-T cells if severe toxicity occurs [76, 77].

Based on the biologic properties of exosomes, CAR-T cell-derived exosomes hold great potential to be used as direct attacker for CAR-T cell-mediated cancer therapy, thereby making these “living drugs” practically controllable. To a certain extent CAR-T cell-induced toxicity can be controlled by using CAR-T cell-derived exosomes as ultimate tumor attacker to replace the immune cells. This hypothesis is supported by the early work of Zitvogel and colleagues [50]. In their *in vivo* study, exosomes that were produced by mouse DCs pulsed with tumor peptides resulted in the rejection of established tumors. *In vitro*, the exosomes produced by DCs were as potent as similar numbers of DCs (between 0.5 × 10^6^ and 1 × 10^6^ DCs) for the induction of antitumor immune responses. The early studies of Peters *et al* verified the essential role of human T cell-derived exosomes in cytotoxic T lymphocyte (CTL)-target cell interaction [78-80]. The presence of CTL surface membrane molecules (T cell receptor, CD3 and CD8) in CTL-derived exosomes ensures the unidirectional delivery of the lethal hit to targeted tumor cells. When target cell recognition occurs due to the interaction of T cell receptor (TCR) with the proper antigen/MHC combination the conjugate formation results in target cell death [78]. The target cell killing effect is actualized through the lethal compounds in the exosomes including perforin, granzymes and lysosomal enzymes [79]. The production of CTL-derived exosomes is promoted by TCR activation and the presence of TCR/CD3/ζcomplex has also been proved in the membrane of human CTL-derived exosomes in another related study [8]. Although the detailed analysis of CAR-T cell-derived exosomes is currently anticipated, the antigen-targeted cytotoxicity is highly expected to be carried out by CAR-T cell-derived exosomes mainly owing to the targeting molecules on their membrane surface and cytotoxic molecules in their intraluminal content.

### CAR-T cell-derived exosomes as direct attackers paving the way for solid tumor therapy

The adoptive transfer of CAR-T cells has exhibited less satisfying therapeutic effects in solid tumors than in lymphoid maliganancies [81, 82]. In addition to the specificity of tumor antigen used for CAR construction, the difference of microenvironments involving various types of cancer is a major factor interfering with anticancer function of CAR-T cells. The cell-to-cell contact between CAR-T cells and tumor cells is an essential prerequisite for this anticancer strategy, so that CAR-T cells must penetrate strom-rich matrixes of the solid tumor which is unlike lymphoid maliganancies. The microenvironment of solid tumor interferences with the efficacy of CAR-T cells *via* at least two mechanisms. First, active tumor-mediated immunosuppression may limit the activity of CAR-T cells [83]; second, functional changes in T lymphocytes after their *ex vivo* manipulation may lead to the reduced ability of CAR-T cells to penetrate the extracellular matrix (ECM) of a solid tumor. A recent study conducted by Caruana *et al.* provided direct evidence supporting the aforementioned mechanism [84]. They engineered CAR-T cells to express the enzyme heparanase (HPSE), which degrades heparan sulfate proteoglycans, the main components of ECM. HPSE CAR-T cells demonstrated their improved capacity to degrade the ECM thereby promoting tumor T cell infiltration and ultimately anticancer activity. Nevertheless, the application of CAR-T cell-derived exosomes as direct attackers is capable of simplifying CAR-mediated anticancer strategy because of their nanoscaled size.

A key requisite of CAR-T cell-derived exosomes as direct attackers to replace CAR-T cells for anticancer therapy would be their capacity to be located at specific antigen-targeted tumor sites and to attack the tumor cells. Unlike the cell-based approach, in which therapeutic cells are able to actively migrate to the target site [85, 86], cell-free exosomes are small nanometer-sized particles and could be delivered through the blood circulation and other biological fluids as evidenced by the abundance of exosomes found in most biological fluids. Exosomes have the ability to cross biological barriers such as the blood-brain barrier (BBB) [25, 46, 87] and blood-tumor barrier (BTB) as corroborated by the extensive presence of tumor cell-derived exosomes in bodily fluids. The enhanced retention effect and leaky vasculature of the tumor also prompt intravenously injected exosomes to be trafficked to the tumor [88]. Apparently, exosomes are passively delivered everywhere in the body, but their target-oriented distribution is mainly determined by the presence of tissue-specific receptors on their surface originating from parent cells [87]. Their tumor-killing effects are actualized by the transfer of exosome's content to target cells that arises through direct contact between the exosome and the cell membrane, either by surface receptors, by fusion of the two membranes, or by endocytosis [24].

### The targeting specificity of CAR-T cells can be preserved in CAR-T cell-derived exosomes

The targeting specificity of CAR-T cells is determined by an antibody-derived single-chain variable fragment (scFv) in the CAR structure. In terms of therapeutic modality using CD19-CAR-T cells to treat CD19-positive hematological malignancies, the targeted attacking characteristic depends on CD19-specific scFv. The so called “on-target, off-tumor” effect is mainly due to the expression of the same antigen in normal organ or tissues. During the biogenesis of exosomes, cellular membrane proteins, including cell target-related proteins, can be transferred to exosomes as exosomal proteins. Recent studies conducted by Muntasell *et al.* showed that in a 24 hour period, ∼12% of the surface-bound peptide-MHC II complex in B lymphocytes is endocytosed, trafficked to MVBs and released on exosomes [11]. Presumably, the targeting feature of parent cells can be carried forward by their exosomes. This perspective has been shown by a variety of pre-clinical studies, although the evidence on CAR-T cell-derived exosomes is still anticipated. A direct demonstration of targeted exosomes was provided by Alvarez-Erviti *et al.* [87]. Through engineering the dendritic cells to express Lamp2b, an exosomal membrane protein, fused to the neuron-specific rabies viral glycoprotein (RVG) peptide, they obtained mouse brain-targeted exosomes and succeeded in using these exosomes as vehicles to deliver siRNA to the brain. Their neuron-specific distribution after systemic delivery is determined by the presence of RVG that serves as a ligand and specifically binds to the acetylcholine receptors in neurons. Recently, a mesenchymal stem cell (MSC)-mediated anticancer strategy has been intensively studied mainly due to MSCs' tropism toward primary and metastatic tumor locations [86, 89]. The prerequisites for MSCs' tumor-directed homing capability include tumor-derived biological factors and the presence of corresponding receptors in MSCs. Tumors can be characterized as ‘wounds that never heal’, which serves as a continuous source of cytokines, chemokines and other inflammatory mediators. On the other hand, MSCs express receptors for a number of growth factors including PDGF and IGF-1, and chemokine receptors, such as CCR2, CCR3, CCR4 and CCL5 [90]. It is worth noting that all surface markers, signaling molecules and cell adhesion molecules are also present in MSC-derived exosomes [91], suggesting that exosomes may adopt the homing pattern of the parent cell and acquire the same range of surface receptors and associated binding proteins as their parent cells. This natural tropism emphasizes the importance of choosing the appropriate cell source for derivation of exosomes for organ-specific therapeutic modality [37]. Engineered CARs, however, can provide non-HLA-restricted recognition of cell surface components and do not require antigen processing and presentation by HLA [3, 69]. Therefore, CAR-T is more broadly applicable to HLA-diverse patient populations.

### CAR-T cell-derived exosomes can be modified to accommodate to the complexity and volatility of cancer

Engineered CARs not only render CAR-T cells antigen-specific targeting properties but also promote CAR-T cell expansion and cytokine production. As the most direct attacker, cytokines (mainly interleukines, either IL-2, IL-7, IL-15 or IL-21 under standardized conditions [92]) are immanent but dramatically enlarged within CAR-T cells. However, it is impossible for any given attacker or drug to act on any given cancer efficiently, because cancer tissue is profoundly heterogeneous and cancer cells are highly adaptable, which is the basis for drug resistance. In nearly 50% of all cancer cases, resistance to chemotherapy already exists before drug treatment starts (intrinsic resistance), and in a large proportion of the remaining 50%, resistance develops during treatment (acquired resistance) [93]. All efforts to overcome resistance to chemotherapy so far have failed, owing to the enormous heterogeneity and complex biology of cancer cells which exhibit a wide range of individual variations [94]. This might be the reason why only a certain proportion of patients with CD19^+^ B-cell leukemia were successfully treated with CD19-CAR-T cells [70, 95]. Considering the heterogeneity and variability of cancer, an ideal anticancer strategy should be diversified and capable of acting concurrently. In addition, the anticancer agents can be adaptively replaced corresponding to patient's specific circumstances. Indeed, CAR-T cells and CAR-T cell-derived exosomes provide desirable platforms to actualize aforementioned modifications.

Additional anticancer agents can be added indirectly through CAR-T cells. Exosomes constitute a mechanism for the intercellular communication through delivering various messages from parent cell to acceptor cell in the form of proteins, mRNAs, ncRNAs and miRNAs. The majority of proteins, mRNAs and miRNAs are detected in both exosomes and parent cells [43, 91, 96]. The proteins and RNAs that are transported by exosomes are protected from degradation by extracellular proteases and RNases thereby prolonging their half-life and enhancing their biological activity [24, 43]. Preclinical studies in mice have shown that exosomes isolated from DCs pulsed with tumor peptides could prime specific cytotoxic T lymphocytes *in vivo* and suppress growth of established murine tumors in a T-cell-dependent manner [50, 97], indicating manipulated stimulation-induced signal transfer from parent cell to exosomes. Our recent studies have demonstrated that conditioned media (CM) from anticancer gene TRAIL-engineered MSC (MSC-CM) induced significant cytotoxicity of various human cancer cells, such as HepG2 (liver cancer cell) [98] and ASPC-1(pancreatic cancer cell) [99]. Presumably, MSC-derived exosomes play an important role for the shuttle of anticancer agents from MSCs toward cancer cells. Because this anticancer gene engineering was carried out with TRAIL-bearing expression vectors, the corresponding exosome-mediated TRAIL delivery could be in the form of cDNA, mRNA or protein. Using a myocardial infarction model, Gnecchi *et al.* demonstrated that CM from genetically engineered MSCs cultured *in vitro* have an efficacy comparable to that of cell transplantation in preventing ventricular remodeling [100]. With regard to CAR-T cell-derived exosomes, one or more cancer cell attackers can be indirectly loaded during their biogenesis in *ex vivo* expanded CAR-T cells. Thereafter, combined with intrinsic cytokines of T lymphocytes, multiple anticancer agents can act on cancer cells simultaneously, and the additional attacker can be substituted according to patient's therapeutic condition.

Anticancer agents can also be directly loaded into CAR-T cell-derived exosomes. Since exosomes resemble liposomes consisting of a bi-lipid membrane and an aqueous core, they could potentially be loaded with both hydrophilic and lipophilic agents [101]. For hydrophilic molecules such as mRNA, siRNA and miRNA, exosome loading can be performed by a transient physical (*e.g.* electroporation) or chemical disruption (*e.g.* lipofection) of the exosome membrane. In Alvarez-Erviti *et al.*'s study, exogenous siRNA was directly loaded into DC-derived exosomes by electroporation resulting in strong mRNA and protein knockdown of targeted genes in target cells [87]. For hydrophobic molecules however, exosome can be loaded by a short period of direct co-incubation [102].

Exosomes as nano-carriers make the use of intracellular anticancer gene products more accessible. The majority of anticancer gene products act on tumor cells through intracellular mechanisms, such as P53, Myc and phosphatase and tensin homolog (PTEN). In the field of MSC-mediated gene therapy, MSCs are used as a vehicle to deliver anticancer genes, the intracellular anticancer gene products must be produced in MSCs and secreted into extracellular space and then penetrate into adjacent tumor cells passing through biological membrane, which is a natural barrier for most macromolecules including peptides and proteins. Therefore, a leading sequence and a transacting activator of transcription (TAT) are usually included in the construct of the related expression vectors [103]. However, exosomes have been shown to cross the plasma membrane to deliver their cargo into recipient cells. For example, DC-derived exosomes can deliver peptide-loaded MHC I and II complexes to other DCs and CD4^+^ T cells to regulate immune response [10, 104]. The content in the cytoplasm of parent cells including mRNA and protein can be directly transferred into the cytoplasm of recipient cells bypassing the biological membrane. Theoretically, the anticancer gene-engineered MSCs could deliver anticancer gene products in all possible forms, *i.e.* cDNA, mRNA and protein, through biogenesis of exosomes. This could be the reason why there is no difference between secreting TRAIL and non-secreting TRAIL engineered MSC-induced cancer cell death in our previous study [98]. Apparently, the appropriate application of exosomes will certainly broaden and simplify cell-based gene therapy in the treatment of cancer.

## SUMMARY AND CLINICAL PERSPECTIVES

CAR-based adoptive immunotherapy employs T lymphocytes that are genetically modified to express CARs providing both targeted antigen-binding and T-cell-mediated immune responses. Once infused in the body, CAR-T cells act as “living drugs” constantly exerting cytotoxic attacks on targeted malignant cells. The persistence of their potent tumor-killing capacity depends on their own life-span and further *in vivo* expansion. Whereas, the extent of cytokine release from CAR-T cells and the status of *in vivo* expansion of these cells cannot be appropriately controlled, which is the potential source of adverse events, such as CRS, cytokine storm and “on-target, off-tumor” response. CAR-T cell-derived exosomes hold great therapeutic potential acting as replacements of CAR-T cells which attack tumor cells. Based on the cell-free nature and biological properties, exosomes substitute CAR-T cells as direct attacker for cancer therapy with obvious advantages. First, exosomes can be used as “off-the-shelf” reagents thereby making CRS controllable. Second, due to their nanoscaled size, the appropriate use of exosomes paves the way for CAR-T technology to be applied for solid tumor therapy including targeting tumors in specific regions such as glioblastoma [44, 45]. Third, besides intrinsic cytokines from CAR-T cells, additional attackers can be supplemented into CAR-T cell-derived exosomes to prevent possible resistance, and finally, the combined and/or alternate use of these two platforms (*i.e.* CAR-T cells and CAR-T cell-derived exosomes) will definitely strengthen the application for CAR-based cancer therapy.

**Figure 1:**
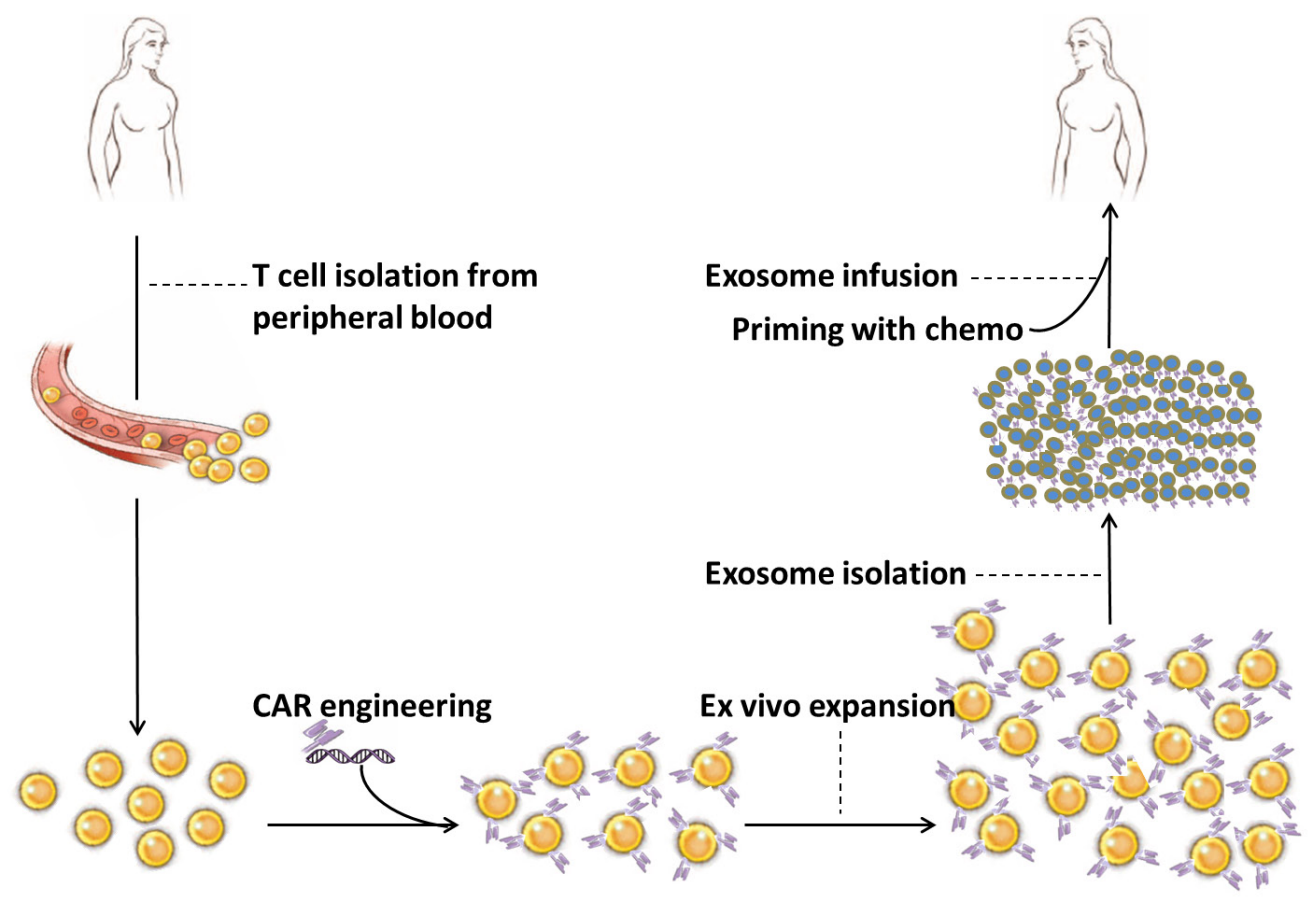
Proposed scheme for the clinical application of CAR-T cell-derived exosomes Peripheral blood sample (30-50 ml) is taken from cancer patient. Total nucleated cells are isolated through density separation or red blood cell lysate solution and cultured with T cell stimulators, such as antibodies for CD3 and CD28. CD8^+^ T cells are selected through positive or negative selection method and transfected with CARs through viral or non-viral transfection technology. CAR-engineered T cells are *ex vivo* expanded in the presence of IL-2. CAR-T cell-derived exosomes are isolated from culture media as described in the text. Exosomes are characterized by biomarker assessment and quantified by protein assay. Exosomes are infused into the same patient after preconditioning with chemotherapy.

As illustrated in Figure 1, the proposed scheme for the clinical application of CAR-T cell-derived exosomes includes collection of T cells from peripheral blood of cancer patient, viral or non-viral insertion of CARs into T cells, *ex vivo* expansion of CAR-engineered T cells, isolation of CAR-T cell-derived exosomes and exosome infusion into the same patient. Based on the special characteristics of the exosomes, such as size (30-150 nm), density (1.1-1.18 g/ml) and evolutionary conserved set of protein molecules (CD9, CD63, CD81, Alix and Tsg101), CAR-T cell derived exosomes can be isolated from culture media through various methods including ultrafiltration [105], ultracentrifugation [10] and affinity capture on antibody-coupled magnetic beads [106]. Other procedures are described in the figure legend. It is definitely warranted to conduct well designed preclinical studies to characterize CAR-T cell-derived exosomes prior to applying them in any clinical setting.

In conclusion, CAR-based T-cell adoptive immunotherapy is a promising therapy for cancer treatment. As a novel and potent therapeutic modality, it is confronting some defects or limitations with regard to the complexity and volatility of cancer. As an extension of this impactful modality, CAR-T cell-derived exosomes may substitute CAR-T cells to act as ultimate attackers, thereby overcoming some of the limitations in current treatment models. The appropriate application of both cellular and exosomal platforms will make this effective treatment more functional, and hopefully bring us closer to developing a novel targeted cancer treatment option.
